# Activation of Adenosine A_3_ Receptor Inhibits Microglia Reactivity Elicited by Elevated Pressure

**DOI:** 10.3390/ijms21197218

**Published:** 2020-09-30

**Authors:** Joana Ferreira-Silva, Inês D. Aires, Raquel Boia, António Francisco Ambrósio, Ana Raquel Santiago

**Affiliations:** 1 Coimbra Institute for Clinical and Biomedical Research (ICBR), Faculty of Medicine, University of Coimbra, 3000-548 Coimbra, Portugal; joanacfsilva@gmail.com (J.F.-S.); inesaires9@gmail.com (I.D.A.); raquelfboia@gmail.com (R.B.); afambrosio@fmed.uc.pt (A.F.A.); 2 Center for Innovative Biomedicine and Biotechnology (CIBB), University of Coimbra, 3000-548 Coimbra, Portugal; 3 Clinical Academic Center of Coimbra (CACC), 3000-548 Coimbra, Portugal; 4 Association for Innovation and Biomedical Research on Light and Image, 3000-548 Coimbra, Portugal

**Keywords:** adenosine A_3_ receptor, elevated hydrostatic pressure, glaucoma, microglia, neuroinflammation

## Abstract

Glaucoma is a progressive chronic retinal degenerative disease and a leading cause of global irreversible blindness, characterized by optic nerve damage and retinal ganglion cell (RGC) death. Elevated intraocular pressure (IOP) is a main risk factor of glaucoma. Neuroinflammation plays an important role in glaucoma. We have been demonstrating that elevated pressure triggers microglia reactivity that contribute to the loss of RGCs. Adenosine, acting on adenosine receptors, is a crucial modulator of microglia phenotype. Microglia express all adenosine receptors. Previously, we demonstrated that the activation of adenosine A_3_ receptor (A_3_R) affords protection to the retina, including RGCs, unveiling the possibility for a new strategy for glaucoma treatment. Since microglial cells express A_3_R, we now studied the ability of a selective A_3_R agonist (2-Cl-IB-MECA) in controlling microglia reactivity induced by elevated hydrostatic pressure (EHP), used to mimic elevated IOP. The activation of A_3_R reduced EHP-induced inducible nitric oxide synthase (iNOS) expression, microglia migration and phagocytosis in BV-2 cells. In retinal microglia, proliferation and phagocytosis elicited by EHP were also decreased by A_3_R activation. This work demonstrates that 2-Cl-IB-MECA, the selective agonist of A_3_R, is able to hinder microglia reactivity, suggesting that A_3_R agonists could afford protection against glaucomatous degeneration through the control of neuroinflammation.

## 1. Introduction

Glaucoma is a main cause of visual impairment and a leading cause of irreversible blindness worldwide [1]. It is described as a group of optic neuropathies that presents common characteristics such as retinal ganglion cell (RGC) loss, thinning of the retinal nerve fiber layer and cupping of the optic disc [2]. Elevated intraocular pressure (IOP) is the main risk factor for disease onset and progression. The current treatments target IOP lowering; however, even upon an efficient lowering of IOP, the disease still progresses in many patients [3].

The mechanisms that trigger glaucomatous degeneration of RGC and optic nerve axons are still unclear, although early inflammatory responses elicited by glial cells take part in the pathological process [4]. Indeed, microglia become reactive early in the course of the disease and have been implicated in the death of RGCs [5,6,7,8,9,10,11]. Exacerbated microglial cell response has been observed in several experimental models of retinal degeneration, including glaucoma [12,13,14,15,16]. Therefore, strategies to control microglia reactivity may halt glaucomatous degeneration.

Adenosine is a purine nucleoside expressed ubiquitously in all tissues. Adenosine signaling has been implicated in apoptosis and inflammation in the central nervous system [17,18]. The actions of adenosine are mediated by four types of G-protein coupled adenosine receptors, named A_1_, A_2A_, A_2B_ and A_3_ receptors. Several studies place the adenosine A_3_ receptor (A_3_R) in the context of inflammatory diseases, such as inflammatory bone loss and lung injury as well as autoimmune and eye diseases [19]. The A_3_R has been described to be expressed in the retina [20], in retinal neural cell cultures [21], including RGCs [22]. Previously, we have described the protective properties of the A_3_R activation in the retina [23]. Indeed, the selective agonist of A_3_R, 2-Cl-IB-MECA prevents the death of retinal cells and affords protection to the retina [23,24]. Moreover, recently it was reported that A_3_R activation stimulates neurite development in RGC cultures and axonal regeneration in an animal model of optic nerve crush [25]. Since microglia are endowed with A_3_R, in this work we studied the potential of A_3_R agonist in controlling microglia reactivity triggered by elevated pressure. Herein, we show that 2-Cl-IB-MECA, the selective A_3_R agonist, controls microglia reactivity, suggesting that, in addition to confer neuroprotection by directly targeting RGCs, A_3_R agonists may confer protection to RGCs in glaucoma by controlling microglia-mediated neuroinflammation.

## 2. Results

Neuroinflammation has a cardinal role in glaucoma and microglial cells have been described as major players for the inflammatory environment, contributing to glaucomatous neurodegeneration [5,9,10]. In order to mimic elevated IOP, the cells were challenged with elevated hydrostatic pressure (EHP) for 4 or 24 h, as we previously demonstrated that EHP triggers microglia reactivity [26].

### 2.1. Effect of EHP in the Expression of A_3_R in BV-2 Cells

**Figure 1:**
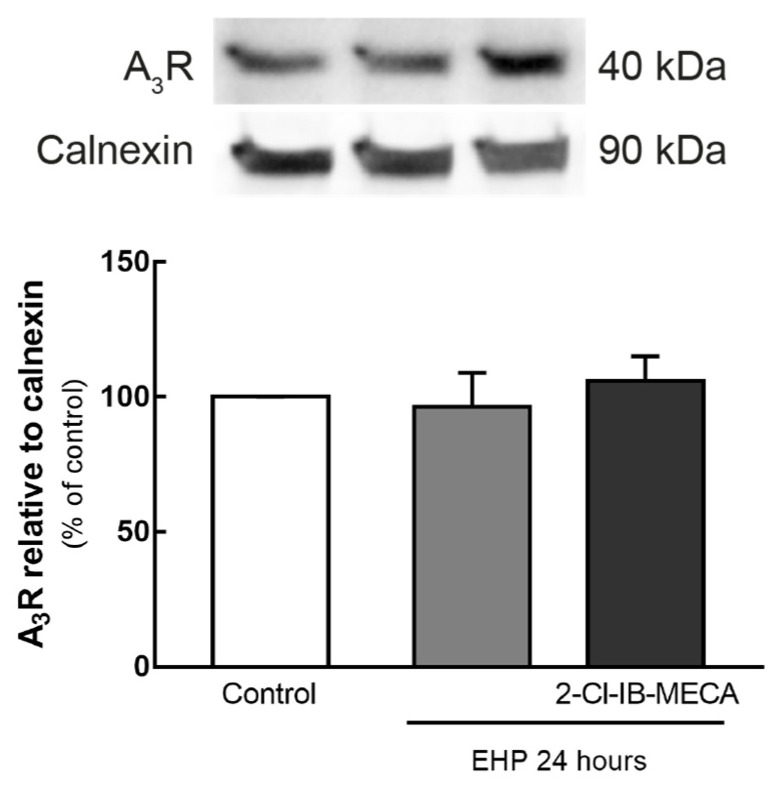
Effect of elevated hydrostatic pressure (EHP) on the expression of adenosine A_3_ receptor (A_3_R) in BV-2 cells. Cells were exposed to EHP for 24 h. A_3_R protein levels were assessed by Western blot and results are expressed as percentage of control from 3 independent experiments. Representative images for A_3_R and calnexin are presented above the graph. Full length uncropped images are presented in Supplementary Figure S1.

BV-2 cells were exposed to EHP for 24 h, in the presence or absence of an A_3_R agonist, 2-Cl-IB-MECA (1 µM), and the protein levels of the receptor were assessed by Western blot (Figure 1). The exposure of BV-2 cells to EHP for 24 h, whether in the presence or absence of the agonist, did not significantly change A_3_R protein levels (96 ± 13% and 106 ± 9% of the control, respectively) when compared with the control.

### 2.2. 2-Cl-IB-MECA Hampers the Increase in iNOS Induced by EHP in BV-2 Cells

**Figure 2:**
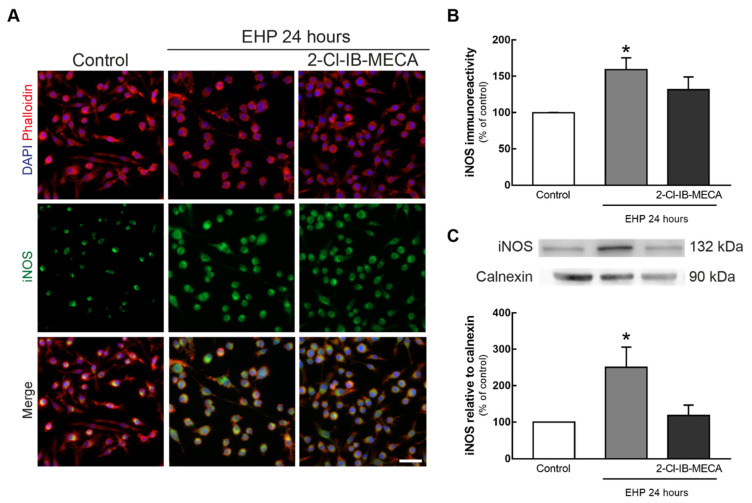
Effect of A_3_R activation in the immunoreactivity and protein levels of inducible nitric oxide synthase (iNOS) in BV-2 cells challenged by EHP. BV-2 cell cultures were incubated with 1 µM 2-Cl-IB-MECA followed the exposure to EHP for 24 h. (**A**) Representative images of microglia labeled with phalloidin (red) and iNOS (green). Nuclei were stained with DAPI (blue). Scale bar: 40 µm. (**B**) Densitometric analyses of iNOS immunoreactivity were determined using Fiji (ImageJ), and results are expressed as percentage of control, from 4 independent experiments. (**C**) iNOS protein levels were assessed by Western blot and expressed as percentage of control from 4–5 independent experiments. Representative images for iNOS and calnexin are presented above the graph. * *p* < 0.05, compared with control, Kruskal–Wallis test, followed by Dunn’s multiple comparison test. Full length uncropped images are presented in Supplementary Figure S2.

BV-2 microglial cells were incubated with 1 µM 2-Cl-IB-MECA and then challenged with EHP for 24 h. The alterations in the density of inducible nitric oxide synthase (iNOS) were evaluated by immunocytochemistry (Figure 2A,B) and the protein levels were assessed by Western blot (Figure 2C). EHP significantly increased iNOS immunoreactivity (Figure 2B) and protein levels (Figure 2C) in BV-2 cells (*p* < 0.05) to 159 ± 16% and 250 ± 55% of the control, respectively. The activation of A_3_R in BV-2 cells exposed to EHP changed the iNOS immunoreactivity and protein levels to 131 ± 17% and 118 ± 29% of the control, respectively.

### 2.3. The Agonist of A_3_R Prevents EHP-Induced BV-2 Cell Migration

**Figure 3:**
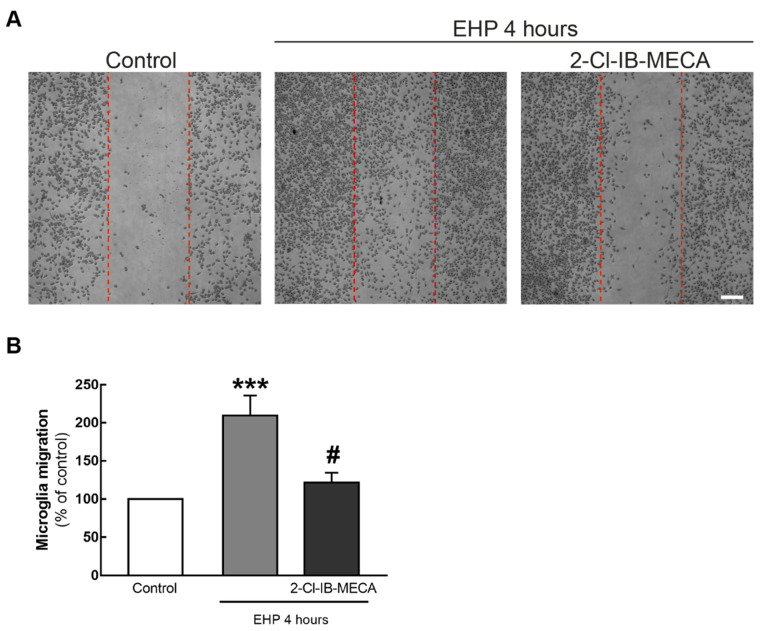
Effect of A_3_R activation in EHP-induced BV-2 cell motility. Cells were pre-treated with 1 µM 2-Cl-IB-MECA followed the exposure to EHP for 4 h. (**A**) Representative images of the cultures at the beginning and at the end of the experiment are depicted. The vertical dotted red lines mark the initial wound. Scale bar: 200 µm. (**B**) The number of cells in the wound was counted per field, and the results were expressed in percentage of control from 9–10 independent experiments. *** *p* < 0.001 compared with control, # *p* < 0.05 compared with EHP, one-way ANOVA test, followed by Holm–Sidak’s multiple comparisons test.

The scratch wound assay was used to assess BV-2 cell migration (Figure 3) after exposing the cells to EHP for 4 h. In control conditions, 222 ± 45 cells migrated and when the cells were exposed to EHP, the number of cells in the wound significantly increased (210 ± 26% of the control; *p* < 0.001) when compared with control. The incubation with the A_3_R agonist significantly (*p* < 0.05) prevented the effect of EHP (122 ± 13% of the control).

**Figure 4:**
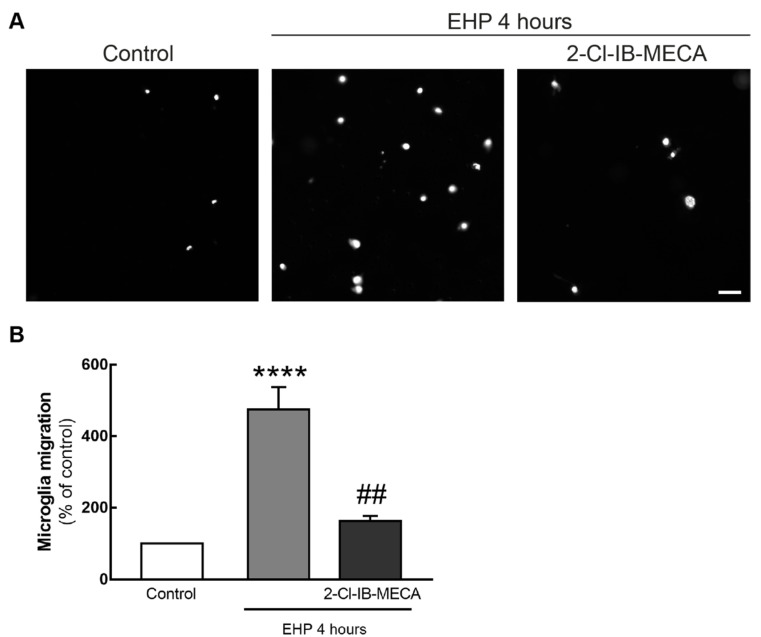
Effect of A_3_R activation on EHP-induced BV-2 cell migration. BV-2 cell migration was assessed by Boyden chamber migration assay. Cells were pre-treated with 1 µM 2-Cl-IB-MECA followed by exposure to EHP for 4 h. (**A**) Representative images are depicted. Scale bar: 50 µm. (**B**) The number of cells in the bottom surface of the transwell was counted and the results are expressed as percentage of control from 5 independent experiments. **** *p* < 0.0001 compared with control, ## *p* < 0.01 compared with EHP, Kruskal–Wallis test, followed by Dunn’s multiple comparison test.

Furthermore, BV-2 cell migration was assessed using a modified Boyden chamber assay (Figure 4A). In control conditions, 2.8 ± 0.3 cells per field were counted in the bottom side of the membrane. When cells were exposed to EHP, the number of cells in the bottom side of the transwell significantly increased to 475 ± 63% of the control (*p* < 0.0001). The incubation with 2-Cl-IB-MECA prevented microglia migration induced by EHP to 163 ± 13% of the control (*p* < 0.001) (Figure 4B).

### 2.4. The Activation of A_3_R Prevents EHP-Induced Increase in Microglia Phagocytic Efficiency

The effect of the selective A_3_R agonist on the phagocytic efficiency of microglial cells was assessed using fluorescent latex beads. BV-2 cells and primary retinal microglial cells were exposed to EHP for 24 and 4 h, respectively, in the presence or absence of the A_3_R agonist.

**Figure 5:**
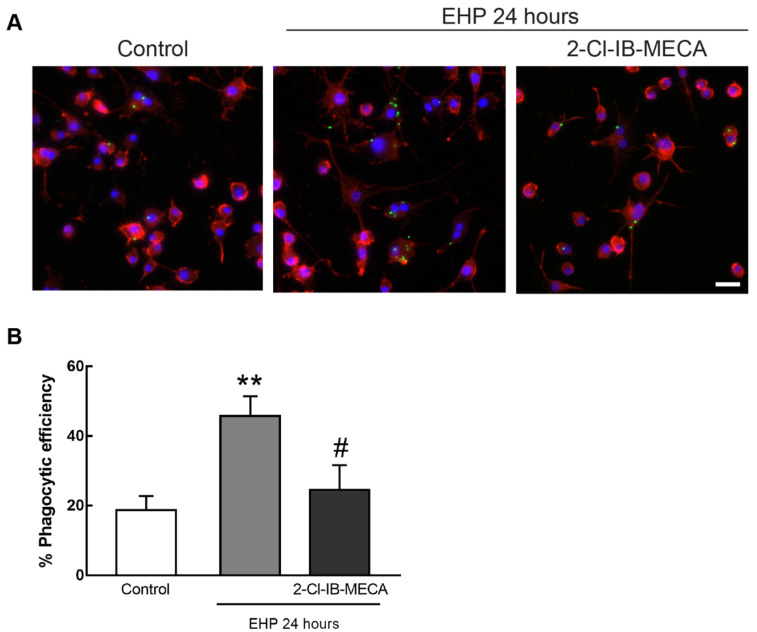
Effect of A_3_R activation in EHP-induced phagocytic activity of BV-2 cells. Cells were incubated with 1 µM 2-Cl-IB-MECA and then exposed to EHP for 24 h. Phagocytosis was assessed with fluorescent latex beads. (**A**) Representative images of BV-2 cells stained with phalloidin (red) with incorporated beads (green). Nuclei were stained with DAPI (blue). Scale bar: 50 µm. (**B**) Phagocytic efficiency was calculated from 4–8 independent experiments. ** *p* < 0.01 compared with control, # *p* < 0.05 compared with EHP, one-way ANOVA test, followed by Holm–Sidak’s multiple comparisons test.

In BV-2 cells (Figure 5A), the phagocytic efficiency was 19 ± 4% in control conditions. The exposure to EHP significantly increased the phagocytic efficiency to 46 ± 6% when compared with control (*p* < 0.01). The pre-incubation with the A_3_R agonist prevented the increase in phagocytic efficiency induced by EHP when compared with EHP conditions (*p* < 0.05) (Figure 5B).

**Figure 6:**
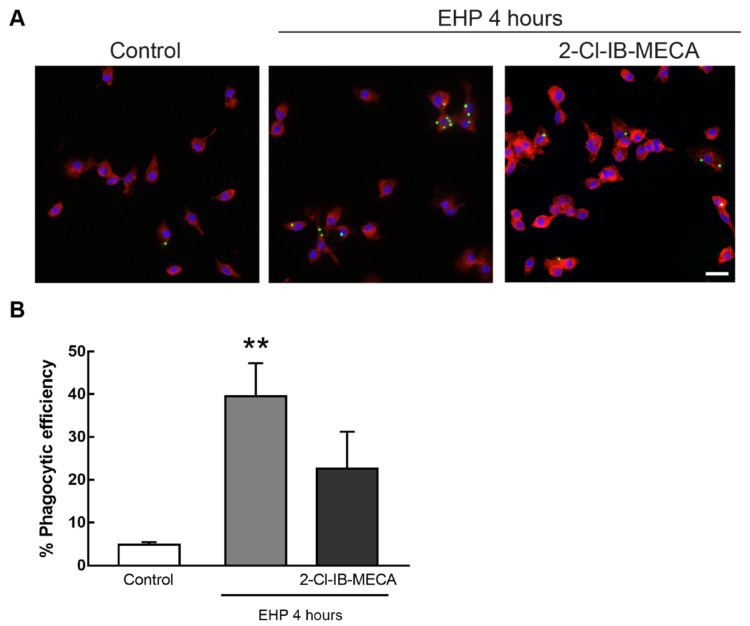
Effect of A_3_R activation in EHP-induced phagocytic activity of primary retinal microglial cells. Cells were incubated with 1 µM 2-Cl-IB-MECA followed by exposure to EHP for 24 h. Phagocytosis was assessed with fluorescent latex beads. (**A**) Representative images of primary microglial cells identified by immunocytochemistry with anti-CD11b (red) with incorporated beads (green). Nuclei were stained with DAPI (blue). Scale bar: 50 µm. (**B**) Phagocytic efficiency was calculated from 4–5 independent experiments. ** *p* < 0.01, compared with control. Kruskal–Wallis test, followed by Dunn’s multiple comparisons test.

In primary retinal microglial cell cultures (Figure 6A), the phagocytic efficiency was 5 ± 0.6% in control conditions. The exposure of retinal microglial cells to EHP significantly increased the phagocytic efficiency to 40 ± 8%, when compared with control (*p* < 0.01). The activation of A_3_R reduced the phagocytic efficiency in EHP conditions to 23 ± 9%, although not significantly (Figure 6B).

### 2.5. The Activation of A_3_R Prevents the Proliferation of Primary Retinal Microglial Cell Elicited by EHP

**Figure 7:**
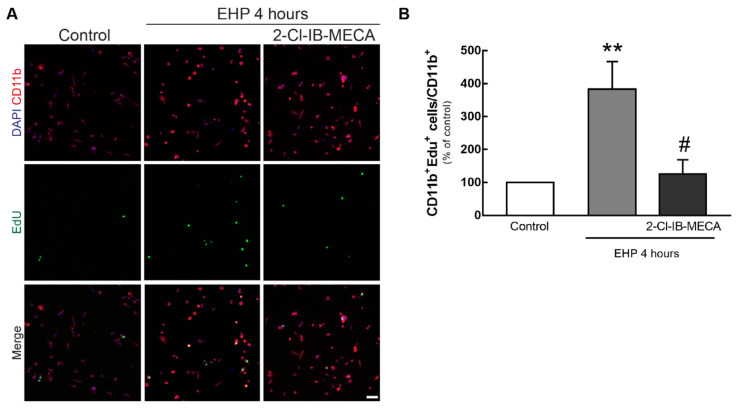
Effect of A_3_R activation in EHP-induced proliferation of primary retinal microglial cells. Cells were incubated with 1 µM 2-Cl-IB-MECA and then exposed to EHP for 4 h. Cell proliferation was measured by counting the number of EdU^+^ cells (green). (**A**) Microglial cells were labeled by immunocytochemistry with anti-CD11b. Nuclei were stained with DAPI (blue). Representative images are depicted. Scale bar: 50 µm. (**B**) The number of proliferating microglial cells (EdU^+^CD11b^+^) was counted, normalized to the total number of microglia and expressed as percentage of the control from 5 independent experiments. ** *p* < 0.01, compared with control, # *p* < 0.05, compared with EHP, Kruskal–Wallis test, followed by Dunn’s multiple comparisons test.

The proliferation of primary retinal microglial cells was evaluated by incubating the cells with a nuclear marker of cell division (EdU) (Figure 7A). When exposed to EHP, the number of proliferating clusters of differentiation molecule 11b (CD11b) positive cells significantly increased to 383 ± 83% of the control (4.1 ± 1.6 CD11b^+^ EdU^+^ cells per field), when compared with the control (*p* < 0.01). The presence of 2-Cl-IB-MECA abolished the effect of EHP on microglia proliferation (125 ± 43% of the control, *p* < 0.05 when compared with EHP) (Figure 7B).

## 3. Discussion

The major risk factor for the development and progression of glaucoma is elevated IOP. The current treatments are focused in IOP lowering; however, in several patients with controlled IOP the disease still progresses to visual field loss [3]. This reinforces the idea that the neuroprotection of RGCs is a valuable strategy to treat glaucoma. We have demonstrated the contribution of microglia-mediated neuroinflammation to the loss of RGCs in experimental models of glaucoma [9,10,12,26], suggesting that targeting both inflammation and RGC neuroprotection would be beneficial to halt disease progression.

The A_3_R is expressed in RGCs [22] and the agonist 2-Cl-IB-MECA promotes neurite development in cultured RGCs and axon regeneration in an animal model of optic nerve crush [25]. Recently, we identified A_3_R as a molecular target with therapeutic potential to protect RGCs [23]. Moreover, we showed that the activation of the A_3_R is neuroprotective to retinal cells, including RGCs, from excitotoxic insults [24]. Furthermore, the intravitreal injection of 2-Cl-IB-MECA provides protection to RGCs against the damage induced by transient retinal ischemia, partial optic nerve transection and ocular hypertension [23,24].

Neuroinflammation mediated by reactive microglia plays an important role in retinal neurodegeneration [5]. Since microglial cells also express the A_3_R [27], and given the role of A_3_R in inflammation [19], we studied whether 2-Cl-IB-MECA could control microglia reactivity elicited by EHP, mimicking elevated IOP.

Elevated pressure did not change the protein levels of A_3_R in BV-2 cells, as we previously reported [28], suggesting that the expression of this receptor is not controlled by elevated pressure as is the expression of A_1_R [28] and A_2A_R [26]. Moreover, the presence of the A_3_R agonist prior exposing the cells to EHP did not modify the protein levels of A_3_R. A previous work demonstrated that A_3_R expression is downregulated upon prolonged agonist exposure (1–24 h) in human astrocytoma cells [29]. This may not be contrary to our findings since in such work the cells were not subjected to noxious conditions [29], as in the current study where the cells were challenged with EHP. Moreover, the extracellular levels of ATP and adenosine (the endogenous ligand of A_3_R) are increased in EHP [28]. Probably, the increase in pressure triggers other mechanisms that would hamper A_3_R downregulation when in the presence of the agonist.

An anti-inflammatory role for A_3_R agonists has been described [30]. LPS-induced increase in TNF levels is inhibited not only in macrophage RAW 264.7 cells after incubation with 10 µM of the A_3_R agonist N6-(3-Iodobenzyl)adenosine-5′-N-methyluronamide (IB-MECA) [31], but also in BV-2 microglial cells after the incubation with 1 µM 2-Cl-IB-MECA [32]. Moreover, evaluation of mRNA and protein levels of iNOS, TNF and IL-Iβ in RAW 264.7 cells revealed that the treatment with the A_3_R agonist 2-chloro-N6-(3-iodobenzyl)-4′-thioadenosine-5′-N-methyluronamide (thio-Cl-IB-MECA) suppresses LPS-induced production of these pro-inflammatory biomarkers in a dose dependent manner (5–20 µM) [33]. Furthermore, a recent study demonstrated that the activation of A_3_R with 0.1 mg/kg/day IB-MECA reduces neuropathic pain by suppressing increased microglia activation [34]. The activation of A_3_R prevents LPS-induced hypoxia-inducible factor 1-alpha (HIF-1α) accumulation, which results in the downregulation of inflammation-associated genes like iNOS [35]. In BV-2 cells, EHP increased iNOS in accordance with the findings of increased nitric oxide synthase 2 (Nos2, inducible) transcript levels in retinal microglial cells exposed to EHP [10]. The A_3_R agonist reduced iNOS protein levels, similar to previous reports demonstrating iNOS expression inhibition by A3R activation [33,36].

When activated, microglial cells proliferate and migrate towards the injury site [5]. Microglia migration can be triggered by different mechanisms involving cytokines, chemokines and growth factors [37]. In BV-2 cells, EHP increases microglia migration and this process was shown to require adenosine and ATP [28], suggesting the involvement of purinergic receptors. The activation of A_3_R prevented EHP-induced microglia migration, in accordance with a previous work demonstrating that A_3_R activation inhibits MCP-1-induced microglial migration [38]. Others have shown that the activation of A_3_R is required for ADP-induced P2Y12-mediated migration of microglia [39]. However, the expression of P2Y12 is drastically reduced in activated microglia [40]. Most likely, since EHP triggers BV-2 microglia reactivity [9,26,28], P2Y12 is downregulated in EHP conditions and not able to control migration. Cell migration may occur by three mechanisms, according to the presence or absence of chemical stimuli: basal motility that takes place in the absence of a chemical stimulus; chemokinesis, characterized by random motility in response to a chemical stimulus; and chemotaxis, in which cells migrate towards a concentration gradient [41,42]. Microglia migration relies on the organization of the cell actin cytoskeleton that is controlled by Rho GTPases [43]. Interestingly, the activation of A_3_R influences the polarized expression of Rho GTPases in response to noxious stimuli [38], leading to the loss of direction in microglia, suggesting chemotaxis as the mechanism by which A_3_R activation is preventing EHP-induced microglia migration.

Another feature of retinal microglia in the adult retina during stressful or pathological conditions is to phagocyte damaged cells, pathogens and cellular debris [44,45]. The exposure of microglia to EHP increased their phagocytic efficiency, as demonstrated previously [26]. In BV-2 cells and primary retinal microglia cell culture, this effect was abolished by the A_3_R agonist, showing that 2-Cl-IB-MECA is able to control microglia phagocytosis.

In addition, responsive microglia also present increased proliferation rate [46]. Indeed, in noxious conditions, microglia proliferate in order to interact with damaged cells [47]. In the DBA/2J glaucoma mouse model, retinal microglial cells proliferate in RGC surroundings in response to glaucomatous damage [48]. It was also reported that there is an increase in microglia proliferation in the retina induced by N-methyl-D-aspartate (NMDA) [49]. In accordance with our previous work, EHP increases the proliferation of retinal microglial cells [26], and the presence of 2-Cl-IB-MECA prevented EHP-induced microglia proliferation, further indicating that A_3_R activation controls microglia reactivity.

This work demonstrates that the activation of A_3_R is able to control microglia reactivity elicited by elevated pressure. This is an in vitro study in which the contribution of other cells is not considered, but future studies evaluating whether A_3_R agonists can control microglia-mediated neuroinflammation in an animal model of ocular hypertension will help clarifying the potential anti-inflammatory effects of A_3_R activation. Taken these results showing that 2-Cl-IB-MECA reduces pro-inflammatory microglia responses elicited by elevated pressure and our previous reports [23,24], one may envisage that A_3_R activation may have protective properties to RGCs in glaucomatous conditions, not only by triggering survival mechanisms directly on A_3_R-expressing RGCs, but also by controlling microglia reactivity.

## 4. Materials and Methods

### 4.1. Microglial Cell Line

The microglia BV-2 cell line (ICLC, Genova, Italy) was cultured in Roswell Park Memorial Institute (RPMI) (GIBCO, Life Technologies, Thermo Fisher Scientific, Waltham, MA, USA) medium supplemented with 10% heat inactivated fetal bovine serum (FBS), 100 U/mL penicillin, 100 μg/mL streptomycin (1% Pen/Strep; GIBCO, Life Technologies, Thermo Fisher Scientific, Waltham, MA, USA) and 2 mM L-glutamine, and maintained at 37 °C in a humidified atmosphere of 5% CO_2_. Cells were subcultured every 2–3 days.

For experiments, cells were cultured in RPMI supplemented with 2% FBS, 1% Pen/Strep and 2 mM L-glutamine, and plated at a density of 1.8 × 10^4^ cells/cm^2^ in 6 well plates, 1 × 10^4^ cells/cm^2^ in 12 well plates or 3.8 × 10^4^ cells/cm^2^ in 48-well plates.

### 4.2. Primary Retinal Microglial Cell Cultures

Retinal microglial cell cultures were prepared from mixed retinal cultures obtained from 4 day old Wistar rats, as we described previously [26]. Retinal cells were cultured in 75 cm^2^ flasks in DMEM/F12 supplemented with 10% FBS and 1% Pen/Strep and maintained at 37 °C in a humidified atmosphere of 5% CO_2_. The culture medium was replaced after a week to remove dead cells. Then, after 12–15 days, generally when cells were 80–90% confluent, the flasks were agitated at 110 rpm for 1 h at 37 °C to detach microglia from the mixed culture. The supernatant was collected and centrifuged at 1100 g for 10 min. Microglial cells were then plated at a density of 5.3 × 10^4^ cells/cm^2^ in 24 well plates with glass coverslips pre-coated with poly-D-lysine (0.1 mg/mL). The purity of the culture (98%) was assessed by immunocytochemistry with anti-CD11b [26].

### 4.3. Cell Treatment

The cells were challenged with EHP (70 mmHg above atmospheric pressure) for 4 or 24 h, as previously detailed [10,26,28]. Microglia cultures were incubated with 1 µM 2-chloro-N6-(3-iodobenzyl)-adenosine-5′-N-methyluronamide (2-Cl-IB-MECA, Tocris Bioscience, Cambridge, UK), an A_3_R selective agonist 45 min before exposing the cells to EHP.

### 4.4. Immunocytochemistry

The cells were washed with warm phosphate-buffered saline (PBS, in mM: 137 NaCl, 2.7 KCl, 10 Na_2_HPO_4_, 1.8 KH_2_PO_4_, pH 7.4), fixed with 4% paraformaldehyde (PFA) with 4% sucrose for 10 min and washed again. After fixation, the cells were permeabilized for 5 min with 1% Triton X-100 prepared in PBS and blocked for 30 min with 3% bovine serum albumin (BSA) and 0.2% Tween-20 prepared in PBS. The cells were then incubated with primary antibodies (Table 1) for 90 min and rinsed with PBS. Next, cells were incubated with the secondary antibodies (Table 1) for 60 min, washed with PBS and then incubated with 4′,6-diamidino-2-phenylindole (DAPI, 1:2000; Sigma-Aldrich, Saint Louis, MO, USA) for 10 min. BV-2 cells were also incubated with phalloidin conjugated to tetramethylrhodamine B isothiocyanate (TRITC) (1:500; Sigma-Aldrich, Saint Louis, MO, USA) for 20 min. Phalloidin selectively binds to F-actin, allowing BV-2 cells visualization. The coverslips were rinsed with PBS and then mounted with Dako^TM^ Fluorescent Mounting Medium (Agilent, Santa Clara, CA, USA).

### 4.5. Densitometric Analysis of iNOS Immunoreactivity

Quantitative analysis of total fluorescence in a cell was performed using ImageJ in images of BV-2 cells immunostained for iNOS. The acquisition settings were maintained within each independent experiment. For each condition, four images were randomly acquired and from each one, ten cells were analyzed. Cell immunoreactivity was calculated using the corrected total cell fluorescence (CTCF) following the formula: CTCF = Integrated density—(area of selected cell * mean fluorescence background reading), as we previously described [26].

### 4.6. Scratch Wound Assay

BV-2 cells were cultured in 6-well plates until approximately 80% confluence. A sterile p200 micropipette tip was used to scratch the monolayer of cells, as we previously described [26]. Bright field images were acquired immediately before and after the scratch and 4 h after EHP exposure. For each condition, five images were randomly acquired along the scratch and the number of cells in the wound was counted.

### 4.7. Boyden Chamber Migration Assay

BV-2 cells were plated in transwell cell culture inserts (8.0 µm pore diameter, Merck Millipore, Burlington, MA, USA) at a density of 5 × 10^4^ cells/cm^2^, and then immediately challenged with EHP for 4 h. At the end of the experiment, the cells in the upper side of the transwell were removed with a cotton swab, and the cells at the bottom side of the insert (cells that migrated) were fixed with 4% PFA with 4% sucrose for 10 min. Nuclei were stained with DAPI (1:2000) to allow cell counting. The membrane was removed from the insert and mounted with Dako^TM^ Fluorescent Mounting Medium (Agilent, Santa Clara, CA, USA) in glass slides. The samples were observed in an inverted fluorescence microscope and eight fields were randomly acquired from each condition. The number of migrated cells was counted.

### 4.8. Phagocytosis Assay

Phagocytic efficiency of BV-2 cells and of retinal microglial cells was assessed with fluorescent latex beads (1 µm diameter; Sigma-Aldrich, Saint Louis, MO, USA) as we previously described [10]. Briefly, cells were incubated with 0.0025% beads solution for 60 min at 37 °C before the end of the experiment. Before cell fixation with 4% PFA in 4% sucrose solution, cells were extensively washed with warm PBS in order to remove beads adherent to plasma membrane (and non phagocytosed). Primary microglial cultures were processed for immunostaining with anti-CD11b antibody as described above, and BV-2 cells were stained with phalloidin (1:500). Nuclei were stained with DAPI (1:2000). Cells were observed with an inverted fluorescence microscope (Axio Observer.Z1, Zeiss, Germany) and seven random fields were acquired from each condition. The number of beads phagocytized by each cell was counted, as well as the number of cells with incorporated beads. Phagocytic efficiency was then calculated: Phagocytic efficiency (%) = [(1* × 1 + 2* × 2 + 3* × 3 … + n* × n)/Total number of cells] * 100, where Xn represents the number of cells containing n beads [10].

### 4.9. Cell Proliferation Assay

Cell proliferation was assessed using the Click-iT^TM^ EdU Alexa Fluor^TM^ 488 EdU proliferation kit according to the instructions provided by the manufacturer (Life Technologies^TM^, Thermo Fisher Scientific, Waltham, MA, USA). Briefly, retinal microglial cell cultures were incubated with 10 µM EdU during the experiment. Cells were rinsed, fixed, permeabilized and blocked, and subsequently incubated with Click-iT^TM^ reaction cocktail for 30 min. Microglial cells were immunostained with anti-CD11b antibody (1:500) and nuclei were stained with DAPI (1:2000), as described above. Preparations were observed with an inverted fluorescence microscope (Axio Observer.Z1, Zeiss, Germany) and from each condition, 8 random fields were acquired. The number of cells double labeled with EdU and CD11b was counted, and the results represent the number of microglia proliferating (CD11b^+^EdU^+^ cells) normalized to the total number of microglia. The results are presented as the percentage of the control.

### 4.10. Western Blot

Cells were washed twice with ice-cold PBS and whole cell extracts were collected in radioimmunoprecipitation assay (RIPA) lysis buffer (50 mM Tris HCl, pH 7.4; 150 mM NaCl; 5 mM ethylenediamine tetra-acetic acid (EDTA); 1% Triton X-100; 0.5% sodium deoxycholate (DOC); 0.1% sodium dodecyl sulfate (SDS); 1 mM dithiothreitol (DTT)) supplemented with complete mini protease inhibitor cocktail tablets and phosphatase inhibitors (10 mM NaF; 1 mM Na_3_VO_4_). The protein lysates were quantified by the bicinchoninic acid (BCA) method. Samples were denaturated by adding 6× concentrated sample buffer (0.5 M Tris, 30% glycerol, 10% SDS, 0.6 M DTT, 0.012% bromophenol blue) and heating for 5 min at 95 °C. Equal amounts of protein (30 µg) were separated by SDS-polyacrylamide gel electrophoresis (SDS-PAGE). Proteins were then transferred electrophoretically to polyvinyl difluoride (PVDF) membranes (Millipore, Billerica, MA, USA). The membranes were blocked for 1 h at room temperature in Tris-buffered saline (137 mM NaCl, 20 mM Tris-HCl, pH 7.6) containing 0.1% Tween-20 (TBS-T) and 5% low-fat milk. Afterwards, the membranes were incubated overnight at 4 °C plus 1 h at RT with the primary antibody (Table 1) in TBS-T with 1% milk, and subsequently washed in TBS-T. Next, membranes were incubated for 90 min at room temperature with secondary antibody (Table 1) prepared in TBS-T with 5% milk and rinsed once again with TBS-T. Protein immunoreactive bands were visualized using an Enhanced Chemiluminescence (ECL) system (ImageQuant^TM^ LAS 500, GE Healthcare Life Sciences, Auckland, New Zealand). The membranes were re-probed for calnexin to control for protein loading, and protein immunoreactivity was visualized using an Enhanced Chemifluorescence (ECF) system (Typhoon FLATM 9000, GE Healthcare Life Sciences, Auckland, New Zealand). Digital quantification of band intensity was performed using Image Studio Lite software (LI-COR Biosciences, Lincoln, NE, USA).

### 4.11. Image Acquisition and Analysis

Imaging was performed in a Zeiss Observer Z.1 inverted microscope equipped with 5× (N-Achroplan, 0.15 M27), 20× (Plan-Apochromat, 0.8 NA) and 40× (LD Plan-Neofluar, 0.6 Corr Ph2 M27) objectives, coupled to a AxioCam HRm camera and Zen Blue 2012 software (all from ZEISS, Oberkochen, Germany). All conditions within an experiment were processed simultaneously and imaging settings (exposure time to LED excitation) were conserved. Images were analyzed using the public domain software Fiji (ImageJ) (https://fiji.sc).

### 4.12. Statistical Analysis

Statistical analysis was performed with Graph Pad Prism 6 software. Results are presented as mean ± SEM. Normality of the data was assessed with Shapiro–Wilk test. Statistical significance was determined using the Kruskal–Wallis test followed by Dunn’s multiple comparison test, or one-way ANOVA test followed by Sidak’s multiple comparison test, as indicated in the figure legends. Statistical significance was considered for *p* < 0.05.

## Figures and Tables

**Table 1:** List of primary and secondary antibodies used in the study.

	Supplier (Cat. No.)	Host	Application	Dilution
**Primary antibodies**				
Anti-iNOS	Santa Cruz (SC-650)	Rabbit	Immunocytochemistry	1:1000
			Western Blot	1:500
Anti-CD11b	Bio-Rad (MCA 275G)	Mouse	Immunocytochemistry	1:500
Anti-A_3_R	Millipore (AB1590P)	Rabbit	Western Blot	1:1000
Anti-Calnexin	Sicgen (AB0041-500)	Goat	Western Blot	1:5000
**Secondary antibodies**				
Alexa Fluor^®^ 488 anti-rabbit IgG (H+L)	Thermo Fisher Scientific (A11008)	Goat	Immunocytochemistry	1:500
Alexa Fluor^®^ 568 anti-mouse IgG (H+L)	Thermo Fisher Scientific (A11004)	Goat	Immunocytochemistry	1:500
Alkaline phosphatase anti-rabbit IgG	Sigma (A3687)	Goat	Western Blot	1:10,000
Alkaline phosphatase anti-goat IgG	Thermo Fisher Scientific (31–300)	Rabbit	Western Blot	1:10,000

## Supplementary Materials

Supplementary materials can be found at https://www.mdpi.com/1422-0067/21/19/7218/s1.

## Abbreviations

A_3_R	Adenosine A_3_ receptor
BCA	Bicinchoninic acid
BSA	Bovine serum albumin
CD11b	Cluster of differentiation molecule 11b
CTCF	Corrected total cell fluorescence
DAPI	4′,6-diamidino-2-phenylindole
DOC	Sodium dexycholate
ECF	Enhanced Chemifluorescence
ECL	Enhanced Chemiluminescence
EDTA	Ethylenediamine tetra-acetic acid
EHP	Elevated hydrostatic pressure
FBS	Fetal bovine serum
HIF-1α	Hypoxia-inducible factor 1-alpha
IB-MECA	N6-(3-Iodobenzyl)adenosine-5′-N-methyluronamide
iNOS	Inducible nitric oxide synthase
IOP	Intraocular pressure
LPS	Lipopolysaccharide
NMDA	N-methyl-D-aspartate
Nos2	Nitric oxide synthase 2, inducible
PFA	Paraformaldehyde
Pen/Strep	Penicillin/Streptomycin
PVDF	Polyvinyl difluoride
RGCs	Retinal ganglion cells
RIPA	Radioimmunoprecipitation assay
RPMI	Roswell Park Memorial Institute
SDS	Sodium dodecyl sulfate
TBS-T	Tris-buffered saline with Tween-20
thio-Cl-IB-MECA	2-chloro-N6-(3-iodobenzyl)-4′-thioadenosine-5′-N-methyluronamide
TRITC	Tetramethylrhodamine B isothiocyanate
