# Supplementary material for: Activation of Adenosine A3 Receptor Inhibits Microglia Reactivity Elicited by Elevated Pressure
Source: Int J Mol Sci. 2020 Sep 30;21(19):7218. doi: 10.3390/ijms21197218 (PMC7582754; doi:10.3390/ijms21197218)
Supplement: Supplementary file 1 [file ijms-21-07218-s001.pdf]

Activation of adenosine A3 receptor inhibits microglia reactivity elicited by elevated pressure

Joana Ferreira-Silva[1,2,3], Inês D. Aires[1,2,3], Raquel Boia[1,2,3], António Francisco Ambrósio[1,2,3], Ana Raquel Santiago[1,2,3*]

[1]University of Coimbra, Coimbra Institute for Clinical and Biomedical Research (iCBR), Faculty of Medicine, Coimbra, Portugal;

[2]University of Coimbra, Center for Innovative Biomedicine and Biotechnology (CIBB), Coimbra, Portugal;

[3]Clinical Academic Center of Coimbra (CACC), Coimbra, Portugal.

[4]Association for Innovation and Biomedical Research on Light and Image, Coimbra, Portugal.

* Correspondence: asantiago@fmed.uc.pt; Tel.: +351-239480226

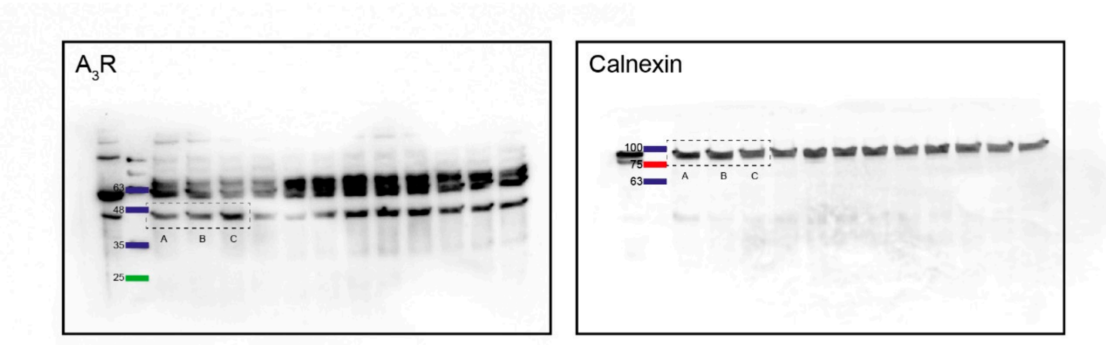

Supplementary Figure 1. Full length Western blots from cropped images depicted in Figure 1. Extracts were collected from BV-2 cells in control conditions (A), from BV-2 cells exposed to EHP for 24 hours (B) and from BV-2 cells treated with 2-Cl-IB-MECA (1 μM) and then exposed to EHP for 24 hours (C).
